# Interactive effect of booster vaccination and vitamin D status on antibody production of Omicron variant‐infected adults: A real‐world cohort study

**DOI:** 10.1111/crj.13694

**Published:** 2023-09-07

**Authors:** Denggao Peng, Yanzhang Gao, Zhichao Liu, Jiawei Zheng, Yingxia Liu

**Affiliations:** ^1^ Graduate Collaborative Training Base of Shenzhen Third People's Hospital, Hengyang Medical School University of South China Hengyang China; ^2^ Department of Emergency Medicine, Shenzhen Third People's Hospital Second Hospital Affiliated to Southern University of Science and Technology Shenzhen China; ^3^ Department of Infectious Diseases, Shenzhen Third People's Hospital Second Hospital Affiliated to Southern University of Science and Technology Shenzhen China

**Keywords:** antibody, booster vaccination, COVID‐19, Omicron, variant, vitamin D

## Abstract

**Introduction:**

Effect of booster vaccination and vitamin D status on antibody production of Omicron variant‐infected adults need to be further explored.

**Methods:**

A retrospective, longitudinal, real‐world cohort study was performed. All included cases were divided into vitamin D deficiency (VDD) and non‐VDD (control) groups according to baseline serum 25‐hydroxyvitamin D [25(OH)D] concentration and then into unvaccinated, routinely vaccinated, and booster vaccinated VDD and control subgroups according to vaccination status. Antibody dynamics were observed within six time periods during hospitalization.

**Results:**

A total of 204 adult cases were included, of which 121 (59%) were males; 23 (11%), 31 (15%), and 26 (13%) or 50 (25%), 35 (17%), and 39 (19%) were unvaccinated, routinely vaccinated, and booster vaccinated VDD cases or controls, respectively. The median (interquartile range) for age and baseline 25(OH)D concentration was 42.5 (31–53.5) years and 21.5 (18–25.4) ng/mL, respectively. The IgM titers within 3 to 7 days and 7 to 14 days increased rapidly to 1.8‐fold (*P* < 0.001) and 3.6‐fold (*P* < 0.001) those within the first day; the IgG titers increased to 5.8‐fold (*P* < 0.001) and 10.9‐fold (*P* < 0.001). Booster vaccinated controls had higher first IgG titers compared with unvaccinated controls (3.1‐fold; *P* = 0.001) or booster vaccinated VDD cases (2.1‐fold; *P* = 0.02).

**Conclusions:**

Booster vaccination and non‐VDD status may have an interactive boosting effect on IgG production of Omicron variant‐infected adults. Further randomized clinical trials may be needed to determine whether booster vaccination combined with VDD correction improves the humoral immunity to Omicron variants.

## Graphical abstract

The SARS‐CoV‐2 IgM and IgG titers increased rapidly within 3 to 14 days during hospitalization in Omicron variant infection. Booster vaccination and vitamin D status may have an interactive boosting effect on IgG production of adult patients with Omicron variant breakthrough infections.

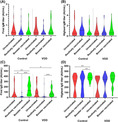

## 1 INTRODUCTION

Since the outbreak of coronavirus disease 2019 (COVID‐19) caused by severe acute respiratory syndrome coronavirus 2 (SARS‐CoV‐2) in late 2019, several variants including Alpha, Beta, Gamma, Delta, and Omicron have been designated by the World Health Organization as variants of concern.^1^ To date, the Omicron variant has become the overwhelmingly dominant strain worldwide with higher transmissibility and partial neutralizing antibody escape.^2^ Unlike its ancestral wild‐type (WT) strain, the Omicron variant has multiple mutations in the antigenic site of the spike protein receptor binding domain (RBD), which could lead to significant changes in antibody production after infection and escape from vaccine‐induced immunity.^3^ Currently, widespread vaccination to build solid immune barriers in populations may be the most critical measure to protect humans from SARS‐CoV‐2 infection. Multiple SARS‐CoV‐2 vaccines, including the most commonly used inactivated virus and mRNA vaccines, have shown efficacy in reducing the severity of COVID‐19.^4^, ^5^ However, these vaccines are basically obtained through multiple technical routes using the WT strain as the immunogen. The protective effect of the vaccine is gradually diminishing as SARS‐CoV‐2 variants continue to evolve through mutations. Unfortunately, breakthrough infections or reinfections have been reported in some vaccine recipients or previously infected individuals, suggesting a possible strong correlation between immune protection and the production of antibodies after vaccination or infection.^6^ Therefore, monitoring antibody responses in Omicron variant‐infected patients may be crucial for epidemic prevention and control, whether naive infections in unvaccinated populations or breakthrough infections in vaccinated or previously infected populations.

As a steroid pro‐hormone that maintains calcium and phosphorus homeostasis, vitamin D has immunomodulatory properties by stimulating the adaptive immune response to shift in the direction of T helper 1 (Th1) or Th2. Typically, the Th1‐type immune response cascades to release pro‐inflammatory cytokines, which may thereby cause high inflammation and even cytokine storms in acute infections.^7^, ^8^ In contrast, the Th2‐type immune response has anti‐inflammatory effects by activating B cell proliferation and maturation, in turn producing pathogen‐specific antibodies.^7^, ^9^ In conditions of vitamin D deficiency (VDD) or insufficiency, the body's immune system shifts in the direction of Th1 and cytokine storm‐induced host tissue damage might occur. Despite some controversy and inconsistency, multiple studies have suggested that vitamin D may reduce COVID‐19 mortality and severity in hospitalized patients, especially in adults with VDD.^10^, ^11^, ^12^ Our previous clinical study found also higher viral loads and lymphocyte depletion in vitamin D‐insufficient children infected with Omicron BA.2, suggesting that vitamin D status may be involved in immunity to Omicron variants.^13^ However, data on the specific effects of vitamin D status after vaccination, especially after booster vaccination, on antibody response to SARS‐CoV‐2 variant breakthrough infections are lacking.

As novel SARS‐CoV‐2 variants continue to emerge, the effectiveness of existing vaccines is being challenged as never before. Although several Omicron‐specific vaccines have been approved for clinical use,^14^ this does not mean that the crisis has been resolved or is going to be resolved soon. There is still an urgent need to understand the regularity of antibody production and improve the immunogenicity and effectiveness of vaccines. The clinical outcome of a COVID‐19 individual largely depends on the host's immunity, especially the production of specific antibodies. Therefore, booster vaccination should be preferred. In addition, vitamin D supplementation for correction of VDD is also one of the important ways to improve host immunity.^12^ In view of the high mutation of SARS‐CoV‐2 variants, the particularity of epidemic prevention and control, and the randomness of its incidence, large‐scale, multi‐center, prospective cohort studies are very difficult to achieve. Therefore, little is known about the interactive effect of booster vaccination and vitamin D status on antibody production. This retrospective, real‐world study may provide data for governments and health service systems to make timely decisions and take appropriate prevention and control measures to combat the challenge of emerging SARS‐CoV‐2 variants.

## 2 MATERIALS AND METHODS

### 2.1 Institutional review board statement

This investigation involving human participants were reviewed and approved by the Ethics Committee of The Third People's Hospital of Shenzhen (approval number: 2022‐100).

### 2.2 Informed consent statement

Written informed consent from patients participating in this study was waived in accordance with the national legislation and the institutional requirements. Patients' personal information will be strictly protected.

### 2.3 Study design and data collection

**FIGURE 1:**
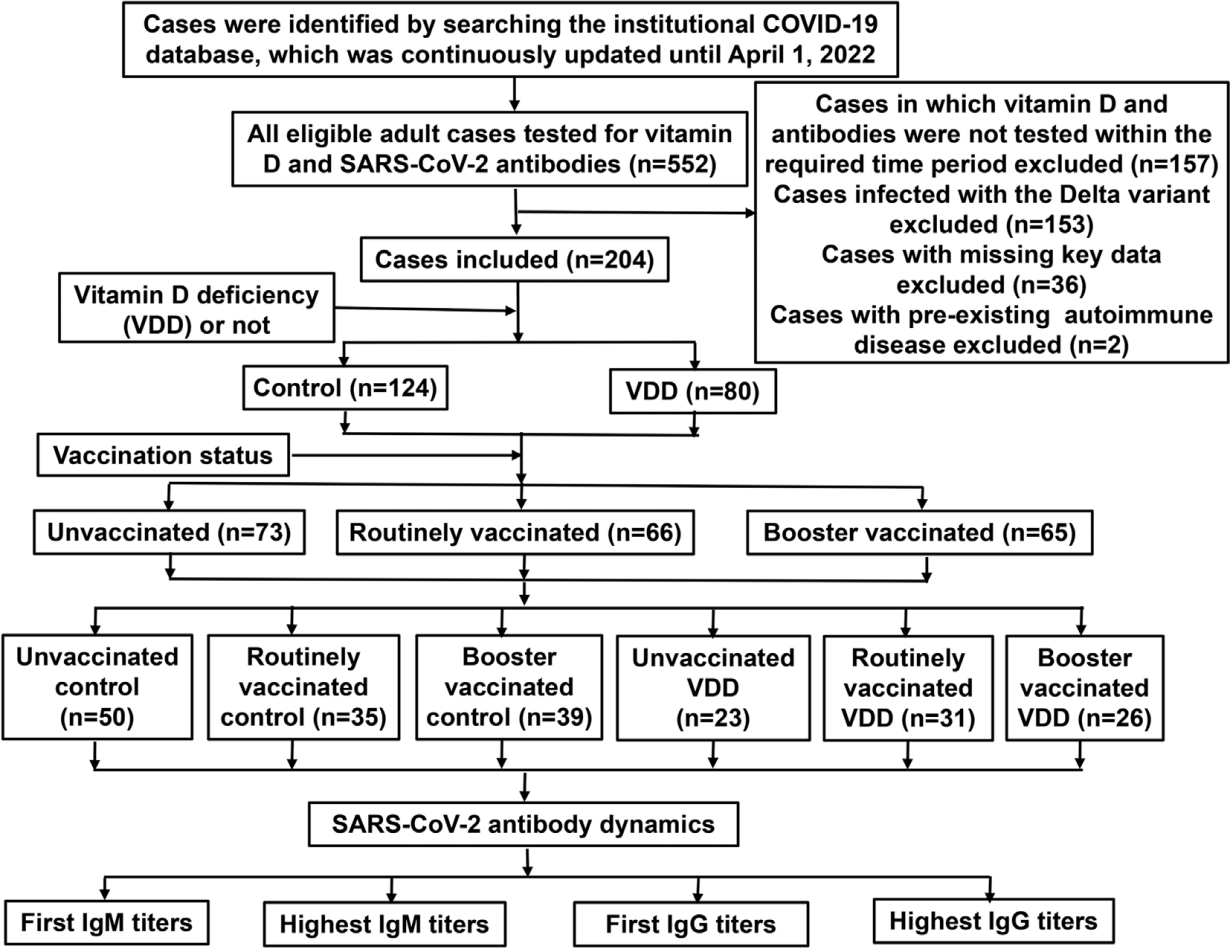
Flow diagram for study design and case selection.

The institutional COVID‐19 database of Shenzhen Third People's Hospital was used, which was continuously updated until April 1, 2022. A retrospective, longitudinal, real‐world cohort study was conducted according to the STrengthening the Reporting of OBservational studies in Epidemiology (STROBE) statement. Our study balanced and controlled confounding factors by stratified design, and multi‐subgroup analysis with appropriate sample size, and directly deleted cases with missing key data. Case selection criteria and data collection form were discussed and developed within the research team before the formal dataset was established, and each team member was trained to ensure data quality. Two authors (JZ and ZL) independently retrieved electronic medical records and identified cases for inclusion based on selection criteria, and a third author (YL) judged and ruled in the cases that consensus was not reached. The clinical and laboratory data of the eligible cases were fully recorded in the data collection form. The authors (DP and YG) participating in the final data analysis were blinded to the grouping settings and conditions. Given that the median (interquartile range, IQR) length of hospital stay was 18 (14–23) days, we selected six time periods from T_1_ to T_6_ during hospitalization to observe antibody dynamics.^14^ T_1_ to T_6_ represent the following: T_1_ ≤ 1 day; 1 < T_2_ ≤ 3; 3 < T_3_ ≤ 7; 7 < T_4_ ≤ 14; 14 < T_5_ ≤ 21; T_6_ > 21 days. The study design and case selection process were presented in Figure 1.

Inclusion criteria:

1. Till April 1, 2022, all discharged adult cases who were infected with the Omicron variant and tested for serum 25‐hydroxyvitamin D [25(OH)D] concentration and SARS‐CoV‐2 antibodies during hospitalization.
2. Given that the adaptive immune system (T and B cells) is fully activated 7 to 10 days after infection, antibody production monitoring was limited to a total of two or more tests, with at least one test each within the first day and 7 to 14 days.

Exclusion criteria:

1. Cases in which the first SARS‐CoV‐2 antibodies and serum 25(OH)D concentrations were not tested within the first day after admission.
2. Cases with missing key data.
3. Previously infected cases.
4. Cases with pre‐existing immunodeficiency, or autoimmune disease, or long‐term oral immunosuppressants.

### 2.4 Clinical definition and classification

Reverse transcription polymerase chain reaction (RT‐PCR) was used to detect SARS‐CoV‐2 positive in nasopharyngeal swab samples. Whole‐genome sequencing and bioinformatics analysis were used to confirm variant types. Fever was recognized when body temperature is higher than or equal to 37.3°C. Respiratory symptoms included nasal congestion, runny nose, sneezing, sore throat, cough, expectoration, chest pain, and dyspnea. Digestive symptoms included nausea, vomiting, abdominal pain, and diarrhea.

### 2.5 COVID‐19 severity

The severity of COVID‐19 were clinically classified according to the Chinese Diagnosis and Treatment Guideline of COVID‐19 (Ninth Trial Version) as follows: (1) asymptomatic, no clinical symptoms; (2) mild, mild clinical symptoms, and no pneumonia on imaging; (3) moderate, obvious clinical symptoms, such as fever, respiratory tract symptoms, pneumonia on imaging; (4) severe, dyspnea or respiratory distress, or respiratory rate (RR) ≥ 30 times/min at rest, or oxygen saturation ≤ 93%, or ratio of partial arterial oxygen pressure (PaO_2_) to fraction of inspired oxygen (FiO_2_) ≤ 300 mmHg (1 mmHg = 0.133 kPa) at standard atmospheric pressure.

### 2.6 Antibody production

The specific IgM and IgG antibodies against both the S1 and N proteins of SARS‐CoV‐2 in serum specimens were quantitatively determined using the Novel Coronavirus (2019‐nCoV) Antibody Detection Kit based on chemiluminescence method according to the manufacturer's instructions (Medical & Biological Laboratories Co., Ltd., China). The observed IgM titers ≥ 10 AU/mL or IgG titers ≥ 10 AU/mL were considered positive, respectively. Antibody seroconversion was defined as the conversion from initial seronegative to first seropositive. The highest IgM and IgG titers were defined as the maximum of two or more test values during hospitalization. If there were two or more measurements in the same time period, their average was calculated.

### 2.7 Vaccination status

Two types of inactivated virus vaccines (BBIBP‐CorV, CoronaVac) commonly used by all vaccinated individuals were manufactured by Sinopharm China Biological Co., Ltd., and Sinovac Biotech Co., Ltd., China. Vaccination status was classified as unvaccinated, routinely vaccinated, and booster vaccinated. The routinely vaccinated was defined as vaccination with a standard two‐dose inactivated virus vaccine. The booster vaccinated was defined as vaccination with an additional third dose of inactivated virus vaccine.

### 2.8 Vitamin D status

Serum 25(OH)D concentration was detected using the vitamin D Total Assay Kit (ADVIA Centaur®, Siemens Diagnostics Inc., USA) based on chemiluminescence method (ADVIA Centaur® XP Immunoassay System). Vitamin D status (deficient or not) was classified into VDD and non‐VDD (control) based on baseline serum 25(OH)D concentration, with reference to cut‐offs commonly used in the guideline: (1) VDD was defined as 25(OH)D concentration < 20 ng/mL; (2) control was defined as 25(OH)D concentration ≥ 20 ng/mL.^15^

### 2.9 Statistical analysis

All analyses were performed using IBM Statistical Product and Service Solutions Version 26 (SPSS 26.0, IBM Inc., Chicago, IL) and GraphPad Prism 8 software. Continuous variables were summarized as median (IQR) or mean ± standard deviation (SD), depending on whether their distributions were normal or not. Categorical variables were summarized as frequency and percentage. Parametric tests (Student's *t*‐test or one‐way analysis of variance) or non‐parametric tests (Mann–Whitney *U* test or Kruskal–Wallis test) for continuous variables and Pearson's chi‐square test or Fisher's exact test for categorical variables were used. *P* values were adjusted with Bonferroni correction for multiple tests. *P* < 0.05 was considered as statistically significant in all tests if applied.

## 3 RESULTS

### 3.1 Demographic and clinical characteristics

Till April 1, 2022, a total of 204 discharged adults with Omicron infection were included in this study, of which 121 (59%) were males; 23 (11%), 31 (15%), and 26 (13%) or 50 (25%), 35 (17%), and 39 (19%) were unvaccinated, routinely vaccinated, booster vaccinated VDD cases or controls, respectively. The median for age and baseline serum 25(OH)D concentration were 42.5 years and 21.5 ng/mL, respectively. There were 36, 155, and 34 cases presented with fever, respiratory symptoms, and digestive symptoms, respectively. There were no severe cases, with six being asymptomatic, 180 being mild, and 18 being moderate. Twenty‐four and five cases had hypertension and diabetes comorbidities. Two cases for IgM and 133 cases for IgG were initially seropositive, respectively. Seroconversion occurred in three and 54 of the 202 and 71 initially seronegative cases, respectively. The median seroconversion interval for IgM and IgG were 7 and 6 days. Detailed demographic and clinical characteristics were presented in Table 1. There were no statistically significant differences in age, male sex, and hypertension comorbidity among the six subgroups (Table S1).

**TABLE 1:** Demographic and clinical characteristics of patients infected with the Omicron variant.

Study population	*n* = 204
Age (years)	42.5 (31–53.5)
Male sex	121 (59%)
Hospital stay (days)	18 (14–23)
Baseline serum 25(OH)D concentration (ng/mL)	21.5 (18–25.4)
Vitamin D deficiency	80 (39%)
Vaccination status	
Unvaccinated	73 (36%)
Routinely vaccinated	66 (32%)
Booster vaccinated	65 (32%)
Severity	
Asymptomatic	6 (3.1%)
Mild	180 (88%)
Moderate	18 (8.9%)
Clinical symptoms	
Fever	36 (18%)
Respiratory symptoms	155 (76%)
Digestive symptoms	34 (17%)
Comorbidities	
Hypertension	24 (12%)
Diabetes	5 (2.5%)
Qualitative SARS‐CoV‐2 IgM	
Initially seropositive	2 (1.0%)
Seroconversion rate	3/202 (1.5%)
Seroconversion interval (days)	7 (6.5–8)
Qualitative SARS‐CoV‐2 IgG	
Initially seropositive	133 (65%)
Seroconversion rate	54/71 (76%)
Seroconversion interval (days)	6 (5–11)

*Note*: The data are presented as the number (%) for categorical variables and the median (IQR) for continuous variables.

Abbreviations: 25(OH)D, 25 hydroxyvitamin D; IQR, interquartile range; SARS‐CoV‐2, severe acute respiratory syndrome coronavirus 2.

### 3.2 Antibody dynamics

**FIGURE 2:**
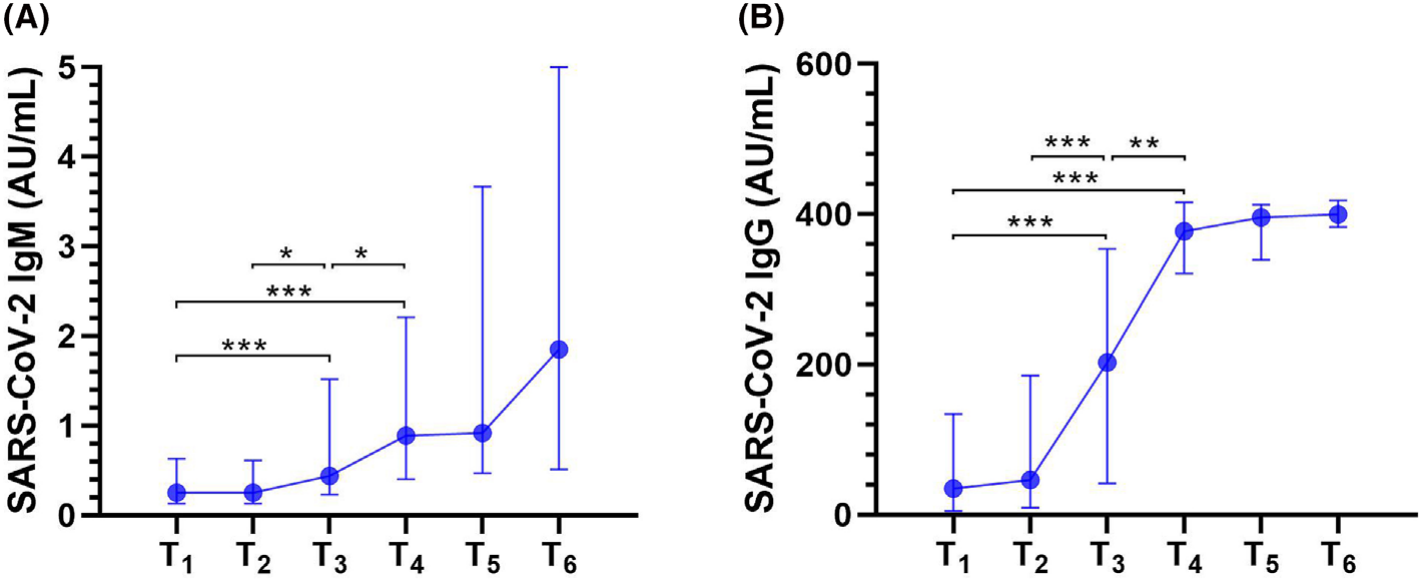
IgM and IgG dynamics within six time periods from T_1_ to T_6_ during hospitalization. T_1_ to T_6_ represent the following: T_1_ ≤ 1 day; 1 < T_2_ ≤ 3; 3 < T_3_ ≤ 7; 7 < T_4_ ≤ 14; 14 < T_5_ ≤ 21; T_6_ > 21 days. Symbols and error bars on the connecting lines represent median with interquartile range, *n* = 204, 93, 124, 91, 31, and 11 within T_1_, T_2_, T_3_, T_4_, T_5_, and T_6_, respectively. *P* values were adjusted with Bonferroni correction for multiple tests. *P < 0.05, **P < 0.01, ***P < 0.001. (A) The IgM titers were significantly higher within T_3_ than T_1_ (1.8‐fold), T_4_ than T_1_ (3.6‐fold), T_3_ than T_2_ (1.8‐fold), T_4_ than T_3_ (2.0‐fold); (B) the IgG titers were significantly higher within T_3_ than T_1_ (5.8‐fold), T_4_ than T_1_ (10.9‐fold), T_3_ than T_2_ (4.4‐fold), and T_4_ than T_3_ (1.9‐fold).

The median (IQR) IgM and IgG titers within T_1_, T_2_, T_3_, T_4_, T_5_, and T_6_ were 0.25 (0.13–0.63) and 34.7 (4.5–134.3), 0.25 (0.13–0.61) and 46.1 (9.1–185.4), 0.44 (0.23–1.52) and 202.5 (41.6–353.6), 0.89 (0.4–2.21) and 377.2 (321.3–415.7), 0.92 (0.47–3.67) and 395.8 (339.3–412.9), and 1.85 (0.51–5) and 399.9 (382.6–418.1) AU/mL, respectively. The IgM and IgG titers were higher within T_3_ than T_1_, T_4_ than T_1_, T_3_ than T_2_, and T_4_ than T_3_, respectively (Figure 2A,B).

### 3.3 Effect of vitamin D status on antibody production

There were 80 (39%) VDD cases and 124 (61%) controls. There were no significant differences in IgM and IgG titers between VDD cases and controls (Table 2).

**TABLE 2:** Effect of vitamin D status on antibody production in Omicron variant‐infected patients.

Antibody titers (AU/mL)	Control (*n* = 124)	VDD (*n* = 80)	*P* value
First IgM titer	0.26 (0.13–0.62)	0.24 (0.13–0.63)	0.64
Highest IgM titer	0.53 (0.29–2.23)	0.56 (0.26–1.55)	0.25
First IgG titer	42.2 (4.9–154.7)	27.8 (3.5–116.2)	0.21
Highest IgG titer	349.9 (166.7–406.7)	338.4 (68.2–394.9)	0.27

*Note*: Antibody titers are presented as median (IQR).

Abbreviations: IQR, interquartile range; VDD, vitamin D deficiency.

### 3.4 Effect of vaccination status on antibody production

There were 73 (36%) unvaccinated, 66 (32%) routinely vaccinated, and 65 (32%) booster vaccinated cases. Compared with the unvaccinated or routinely vaccinated, the booster vaccinated had higher first (2.4‐fold; *P* = 0.002 or 18.9‐fold; *P* < 0.001) and higher highest (2.4‐fold; *P* = 0.002 or 18.9‐fold; *P* < 0.001) IgG titers (Table 3). There was a higher trend in the first IgG titers in patients routinely vaccinated with CoronaVac compared with BBIBP‐CorV, but no statistical difference was observed (Table S2). The first IgG titers were higher in controls booster vaccinated with CoronaVac than with BBIBP‐CorV (Table S3).

**TABLE 3:** Effect of vaccination status on antibody production in Omicron variant‐infected patients.

Antibody titers (AU/mL)	Unvaccinated (*n* = 73)	Routinely vaccinated (*n* = 66)	Booster vaccinated (*n* = 65)	*P* value
First IgM titer	0.29 (0.16–0.94)	0.21 (0.12–0.41)	0.24 (0.15–0.7)	0.09
Highest IgM titer	0.52 (0.27–2.36)	0.65 (0.25–1.55)	0.48 (0.31–1.58)	0.91
First IgG titer	45.7 (3.6–140.6)	5.9 (2.3–19.1)	111.3 (37.7–221.9)	0.002^a^; 0.002^b^; <0.001^c^
Highest IgG titer	309.8 (71.3–393.7）	323.9 (56.3–387.8)	370.9 (320.5–417.8)	1.00^a^; 0.001^b^; 0.003^c^

*Note*: Antibody titers are presented as median (IQR). *P* values were adjusted with Bonferroni correction for multiple tests.

Abbreviation: IQR, interquartile range.

a Unvaccinated versus routinely vaccinated.

b Unvaccinated versus booster vaccinated.

c Routinely vaccinated versus booster vaccinated.

### 3.5 Interactive effect of booster vaccination and vitamin D status

**FIGURE 3:**
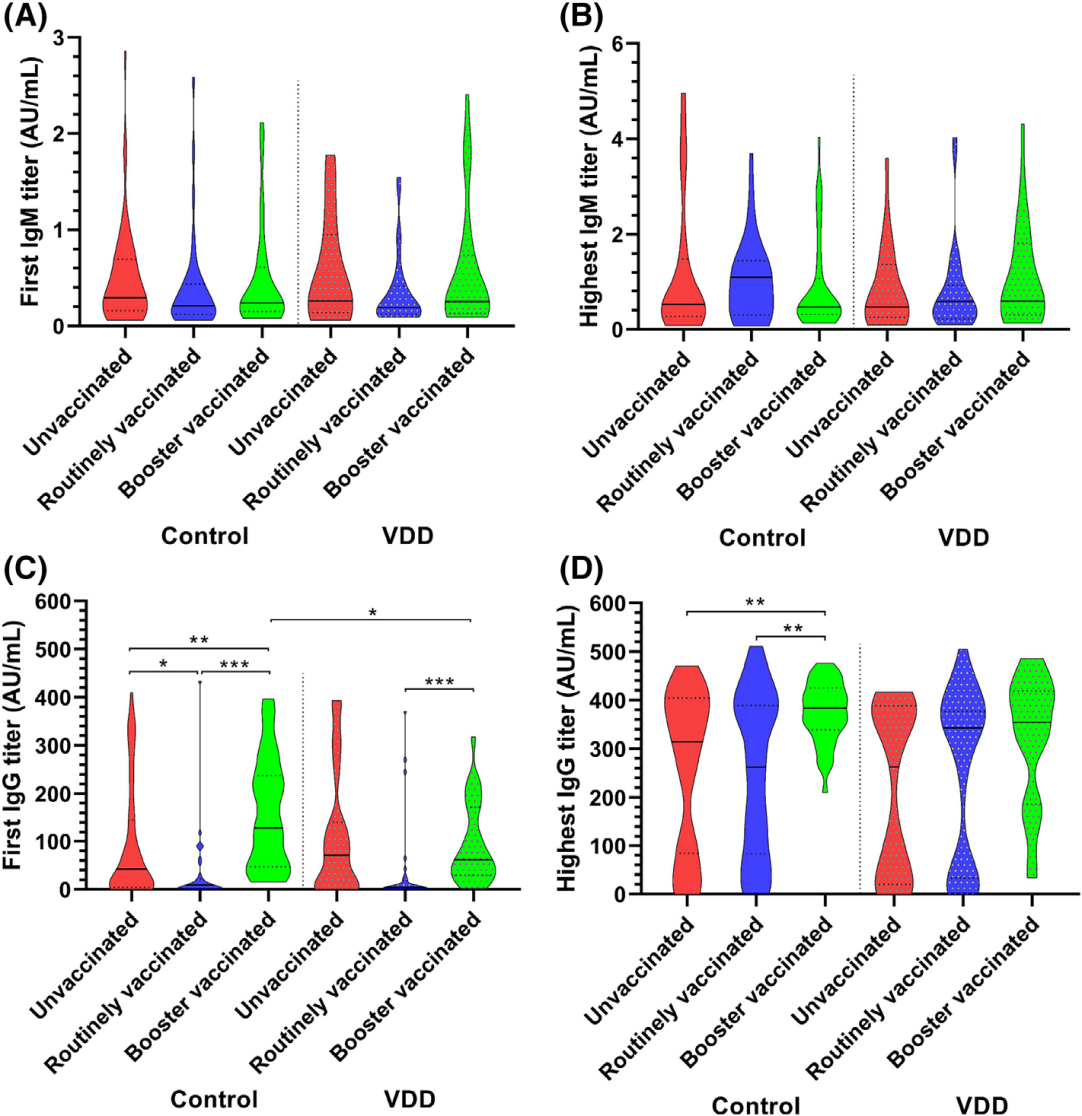
Effect of booster vaccination and vitamin D status on antibody production during hospitalization. Red, blue, or green violin plots represent the unvaccinated, routinely vaccinated, or booster vaccinated, respectively, *n* = 50, 35, 39, 23, 31, and 26 per condition. Solid and dashed lines in the violin plot represent median and quartiles. VDD, vitamin D deficiency. Control, non‐VDD cases. *P* values were adjusted with Bonferroni correction for multiple tests. *P < 0.05, **P < 0.01, ***P < 0.001. (A) First IgM titers; (B) highest IgM titers; (C) first IgG titers; (D) highest IgG titers.

There was no significant difference in IgM titers among the six subgroups (Figure 3A,B). Among VDD cases, the first and highest IgG titers were not statistically different between the booster vaccinated and the unvaccinated. Booster vaccinated controls had higher first IgG (3.1‐fold; *P* = 0.001 or 14.1‐fold; *P* < 0.001 or 2.1‐fold; *P* = 0.02) compared with unvaccinated ones or routinely vaccinated ones or booster vaccinated VDD cases and higher highest IgG titers (1.2‐fold; *P* = 0.005 or 1.5‐fold; *P* = 0.008) compared with unvaccinated ones or routinely vaccinated ones (Figure 3C,D) (Table S4).

## 4 DISCUSSION

To our knowledge, this is the first longitudinal, real‐world cohort study on the interactive effect of booster vaccination and vitamin D status on antibody production of Omicron variant‐infected adults so far. Our observational cohort study highlighted the findings: (1) The IgM and IgG titers of Omicron variant‐infected adult patients increased significantly within 3 to 7 and 7 to 14 days after admission; (2) booster vaccinated controls had 3.1‐fold or 2.1‐fold first IgG titers compared with unvaccinated controls or booster vaccinated VDD cases, respectively, and 1.2‐fold highest IgG titers compared with unvaccinated controls. Through stratified study design and multi‐subgroup analysis with appropriate sample size to control confounding and bias, the final results and the conclusion that booster vaccination and vitamin D status may have an interactive boosting effect on IgG production should be robust and reliable. These informative data could deepen the understanding on the importance of booster vaccination and correction of VDD against Omicron variant infections, which would contribute to the formulation of post‐pandemic booster vaccination strategies.

Multiple studies have reported that IgM and IgG antibodies seroconverted at a median of 12 and 14 days after the onset of symptoms of SARS‐CoV‐2 WT strain infection,^16^ peaked at 2 to 5 weeks and 3 to 7 weeks, and remained detectable for approximately 6 to 8 weeks and at least 12 months, respectively.^17^, ^18^ In addition, clinical factors including COVID‐19 severity as well as demographic factors including age and gender were found to potentially influence antibody responses.^16^, ^19^ Moreover, a recent study presented that the hypertension comorbidity was significantly associated with lower antibody titers in COVID‐19.^19^ However, the antibody response to Omicron infection and its underlying influencing factors remain largely inconclusive.^14^, ^20^ In particular, different vaccination strategies, breakthrough infections, and reinfections have complicated RBD‐specific antibody dynamics.^14^, ^20^ Our study found that IgM and IgG titers increased significantly and rapidly within 3 to 14 days after Omicron infection, with a median seroconversion interval of about 7 days, earlier than those after WT infection. Given that the SARS‐CoV‐2 vaccine was rolled out in late 2020, and the World Health Organization (WHO) named the new variant B.1.1.529 as Omicron on November 26, 2021,^20^ this different antibody dynamics may be related to the immunogenicity of the Omicron variant and the vaccination status. Notably, age, gender, and hypertension comorbidity are clinically unchangeable factors, and it may be more practical and feasible to explore these changeable factors such as vaccination and vitamin D status.

Our previous study have showed that regular and booster vaccination with inactivated virus vaccines enhanced the neutralizing activity against Omicron variants in both the breakthrough infections and vaccinated individual.^20^ In addition, a follow‐up cohort study evaluating antibody immunity to SARS‐CoV‐2 variants by vaccination has suggested that mean plasma IgM and IgG titers increased significantly after the second vaccination and then gradually decreased to low levels over 7 months, and the third vaccination could recall and elicit a stronger IgG response, but not IgM.^21^ However, the present study observed similar but not entirely consistent vaccination‐based antibody responses that the booster vaccinated had higher first and highest IgG titers compared to the unvaccinated or routinely vaccinated, but the routinely vaccinated was not superior to the unvaccinated. Typically, the RBD‐specific antibody response may reflect the immunogenicity of the vaccine, as well as the effectiveness of the vaccination in preventing and fighting infection.^4^, ^21^ More importantly, IgG may play a key role in long‐term immune protection.^4^ These data showed that breakthrough infections with Omicron variants could recall and elicit robust and significant IgG responses based on booster rather than routine vaccination,^20^ highlighting the importance of timely booster following routine vaccination.

Despite some recent and important achievements,^22^, ^23^, ^24^ there are still controversies about the effect of vitamin D status on antibody response after vaccination or infection.^13^, ^22^, ^23^, ^24^, ^25^, ^26^, ^27^, ^28^ A study recruiting 97 healthcare workers found that individuals without VDD had an average of 29.3% higher peak antibody response induced by the first dose of the SARS‐CoV‐2 BNT162b2 vaccine.^23^ Zelini et al. observed a significant correlation between baseline serum 25(OH)D concentration and anti‐S neutralizing antibody response at 6 months after the second vaccination and concluded that adequate vitamin D levels could improve immune responses to mRNA vaccines.^24^ However, other several observational studies investigating the association between vitamin D status and the immunogenicity of SARS‐CoV‐2 vaccines have yielded inconsistent results.^25^, ^26^, ^27^ In addition, higher titers of anti‐S antibodies after vaccination have been reported in individuals taking vitamin D supplements,^27^ but other studies have not found the similar relationship.^25^ Notably, significant differences between studies in demographic characteristics (age and male sex ratio), type of vaccine administered, baseline vitamin D status, and time point for antibody testing could explain the reasons behind these inconsistent results.

Given the limitations of current clinical studies (lack of large multicenter randomized clinical trials, large heterogeneity of the cohort, insufficient size, and representation of the sample, etc.), it is difficult to obtain reliable evidence on the effect of booster vaccination and vitamin D status on antibody production of Omicron variant‐infected adults in the real‐world, especially the interactive effect. Interestingly, our study observed no statistically significant differences in age, male sex, and hypertension comorbidity among the six subgroups and higher first IgG titers in booster vaccinated controls than unvaccinated controls or booster vaccinated VDD cases, suggesting that the booster vaccination and vitamin D status might have an interactive boosting effect on IgG production. Essentially, the first IgG titer primarily represents memory humoral immunity post‐vaccination or post‐infection. It is speculated that non‐VDD status may also affect IgG production after booster vaccination, and correction of VDD to boost immune protection of the vaccine after booster vaccination may be reasonable. These results highlight the importance of detecting vitamin D status and correcting VDD after vaccination or infection.

There are several limitations in our study. (1) Due to the single‐center, retrospective, observational nature, there are certain confounding factors. (2) Due to particularly strict quarantine in China, some asymptomatic or mildly symptomatic patients with Omicron infection were admitted to hospital in the early stages of COVID‐19. Antibody dynamics in our study started from hospital admission, which was different from those in previous studies that start from symptom onset. (3) This longitudinal study observes the antibody dynamics over time periods rather than time points, which may have some influence on the final results. (4) Some patients were not tested for antibodies in every time period, and there was a certain difference between the highest antibody titer and the peak antibody titer. Therefore, these results should be carefully interpreted due to potential selection bias and residual confounding.

## 5 CONCLUSIONS

The SARS‐CoV‐2 IgM and IgG titers increased rapidly within 3 to 14 days during hospitalization. Booster vaccination and non‐VDD status may have an interactive boosting effect on IgG production of Omicron variant‐infected adults. Further randomized clinical trials may be needed to determine whether booster vaccination combined with VDD correction improves the humoral immunity to Omicron variants.

## AUTHOR CONTRIBUTIONS

All authors have read and approved the final manuscript version to be submitted. Denggao Peng was responsible for methodology, investigation, formal analysis, data curation, writing the original draft, and visualization, Jiawei Zheng and Zhichao Liu for investigation and data curation, and Yingxia Liu for conceptualization, investigation, review and editing, and supervision. Yanzhang Gao worked on conceptualization, formal analysis, investigation, and data curation.

## CONFLICT OF INTEREST STATEMENT

The authors declare that the research was conducted in the absence of any commercial or financial relationships that could be construed as a potential conflict of interest.

## ETHICS STATEMENT

This investigation involving human participants was reviewed and approved by the Ethics Committee of The Third People's Hospital of Shenzhen (approval number: 2022‐100). Written informed consent from patients participating in this study was waived in accordance with the national legislation and the institutional requirements. Patients' personal information will be strictly protected.

## Supporting information


**Table S1:** Baseline demographic characteristics among six subgroups of Omicron‐infected patients.
**Table S2:** Effect of type of vaccines on antibody production in routinely vaccinated patients.
**Table S3:** Effect of type of vaccines on antibody production in booster vaccinated patients.
**Table S4:** Interactive effect of vaccination and vitamin D status on antibody production.

## Data Availability

All data generated or analyzed during this study are included in this article. Further enquiries can be directed to the corresponding author.
