# Supplementary material for: Interactive effect of booster vaccination and vitamin D status on antibody production of Omicron variant‐infected adults: A real‐world cohort study
Source: Clin Respir J. 2023 Sep 7;17(10):1067–76. doi: 10.1111/crj.13694 (PMC10542990; doi:10.1111/crj.13694)
Supplement: Supplementary file 1 — Table S1: Baseline demographic characteristics among six subgroups of Omicron‐infected patients. Table S2: Effect of type of vaccines on antibody production in routinely vaccinated patients. Table S3: Effect of type of vaccines on antibody production in booster vaccinated patients. Table S4: Interactive effect of vaccination and vitamin D status on antibody production. [file CRJ-17-1067-s001.docx]

Table S1: Baseline demographic characteristics among six subgroups of Omicron-infected patients

| Vaccination status | Age (years) | | *P* value |
| --- | --- | --- | --- |
|  | Control | Vitamin D deficiency |  |
| Unvaccinated  Routinely vaccinated  Booster vaccinated | 45 ± 13 n=50  41 ± 15 n=35  46 ± 12 n=39 | 40 ± 11 n=23  44 ± 16 n=31  42 ± 13 n=26 | 0.09  0.49  0.25 |
| *P* value | 0.24 | 0.53 |  |

| Vaccination status | Male sex | | *P* value |
| --- | --- | --- | --- |
|  | Control | Vitamin D deficiency |  |
| Unvaccinated  Routinely vaccinated  Booster vaccinated | 27 (54%) n=50  24 (69%) n=35  26 (67%) n=39 | 9 (39%) n=23  19 (61%) n=31  16 (62%) n=26 | 0.08  0.61  0.79 |
| *P* value | 0.31 | 0.19 |  |

| Vaccination status | Hypertension comorbidity | | *P* value |
| --- | --- | --- | --- |
|  | Control | Vitamin D deficiency |  |
| Unvaccinated  Routinely vaccinated  Booster vaccinated | 5 (10%) n=50  7 (20%) n=35  5 (13%) n=39 | 2 (8.7%) n=23  5 (16%) n=31  0 (0%) n=26 | 1.00  0.76  0.18 |
| *P* value | 0.41 | 0.10 |  |

The data are presented as the number (%) for categorical variables and the mean ± SD for continuous variables. *P* values were adjusted with Bonferroni correction for multiple tests. SARS-CoV-2, severe acute respiratory syndrome coronavirus 2; SD, standard deviation.

Table S2: Effect of type of vaccines on antibody production in routinely vaccinated patients

| Type of vaccines | First SARS-CoV-2 IgM titers | | *P* value |
| --- | --- | --- | --- |
|  | Control | Vitamin D deficiency |  |
| BBIBP-CorV  CoronaVac  Others | 0.12 (0.08-0.34) n=13  0.29 (0.14-0.49) n=18  0.37 (0.22-1.1) n=4 | 0.19 (0.12-0.28) n=8  0.16 (0.1-0.42) n=17  0.28 (0.13-0.36) n=6 | 0.28  0.21  0.45 |
| *P* value | 0.09 | 0.68 |  |

| Type of vaccines | Highest SARS-CoV-2 IgM titers | | *P* value |
| --- | --- | --- | --- |
|  | Control | Vitamin D deficiency |  |
| BBIBP-CorV  CoronaVac  Others | 0.36 (0.19-1.58) n=13  1.1 (0.53-1.51) n=18  0.43 (0.25-1.67) n=4 | 0.63 (0.29-2.57) n=8  0.59 (0.18-1.18) n=17  0.33 (0.22-0.47) n=6 | 0.29  0.14  0.67 |
| *P* value | 0.39 | 0.41 |  |

| Type of vaccines | First SARS-CoV-2 IgG titers | | *P* value |
| --- | --- | --- | --- |
|  | Control | Vitamin D deficiency |  |
| BBIBP-CorV  CoronaVac  Others | 2.9 (1.2-6.8) n=13  10.1 (4.6-29) n=18  59.6 (28.9-91) n=4 | 1.7 (0.4-3.2) n=8  5.3 (3.8-11.4) n=17  133.8 (9.2-270.6) n=6 | 0.66  0.16  0.67 |
| *P* value | 0.06*; 0.07**; 1.00*** | 0.06*; 0.005**; 0.46*** |  |

| Type of vaccines | Highest SARS-CoV-2 IgG titers | | | *P* value |
| --- | --- | --- | --- | --- |
|  | Control | Vitamin D deficiency | |  |
| BBIBP-CorV  CoronaVac  Others | 165.4 (78.3-420.6) n=13  311.1 (168-387.8) n=18  144.5 (45.4-278.3) n=4 | 335.6 (165.9-371.6) n=8  343.2 (33.6-415.7) n=17  314.4 (99.2-366.7) n=6 | | 0.61  0.82  0.21 |
| *P* value | 0.47 | | 0.91 |  |

Antibody titers are presented as median (IQR). IQR, interquartile range. *P* values were adjusted with Bonferroni correction for multiple tests. SARS-CoV-2, severe acute respiratory syndrome coronavirus 2. *BBIBP-CorV vs. CoronaVac; **BBIBP-CorV vs. Others; ***CoronaVac vs. Others.

Table S3: Effect of type of vaccines on antibody production in booster vaccinated patients

| Type of vaccines | First SARS-CoV-2 IgM titers | | *P* value |
| --- | --- | --- | --- |
|  | Control | Vitamin D deficiency |  |
| BBIBP-CorV  CoronaVac  Both  Others | 0.23 (0.11-0.25) n=10  0.2 (0.17-0.67) n=16  0.36 (0.27-0.73) n=6  0.24 (0.14-0.48) n=7 | 0.18 (0.11-0.24) n=10  0.39 (0.25-0.91) n=12  0.42 (0.11-1.27) n=4 | 0.85  0.19  0.75 |
| *P* value | 0.33 | 0.21 |  |

| Type of vaccines | Highest SARS-CoV-2 IgM titers | | *P* value |
| --- | --- | --- | --- |
|  | Control | Vitamin D deficiency |  |
| BBIBP-CorV  CoronaVac  Both  Others | 0.4 (0.31-0.56) n=10  0.48 (0.3-1.49) n=16  0.48 (0.4-2.47) n=6  0.49 (0.27-1.3) n=7 | 0.4 (0.25-0.77) n=10  0.81 (0.42-1.93) n=12  0.79 (0.47-1.36) n=4 | 0.79  0.64  1.00 |
| *P* value | 0.69 | 0.41 |  |

| Type of vaccines | First SARS-CoV-2 IgG titers | | *P* value |
| --- | --- | --- | --- |
|  | Control | Vitamin D deficiency |  |
| BBIBP-CorV  CoronaVac  Both  Others | 35.2 (24.4-61.3) n=10  186.5 (82.2-230.2) n=16  199.2 (105.4-297.9) n=6  231.3 (217.7-322.2) n=7 | 32.6 (27-57.6) n=10  116.2 (49.4-218.4) n=12  129.3 (38.2-203.1) n=4 | 0.97  0.55  0.29 |
| *P* value | 0.01*; 0.045**; 1.00*** | 0.09 |  |

| Type of vaccines | Highest SARS-CoV-2 IgG titers | | *P* value |
| --- | --- | --- | --- |
|  | Control | Vitamin D deficiency |  |
| BBIBP-CorV  CoronaVac  Both  Others | 341.1 (300-435.6) n=10  375 (356-408.5) n=16  338 (272.8-442.8) n=6  397.1 (386.4-432.7) n=7 | 320.5 (176.9-400.1) n=10  388.8 (329.2-420.3) n=12  398.7 (284.4-451.7) n=4 | 0.41  0.93  0.67 |
| *P* value | 0.51 | 0.39 |  |

Antibody titers are presented as median (IQR). IQR, interquartile range. *P* values were adjusted with Bonferroni correction for multiple tests. SARS-CoV-2, severe acute respiratory syndrome coronavirus 2. *BBIBP-CorV vs. CoronaVac; **BBIBP-CorV vs. Both; ***CoronaVac vs. Both.

Table S4: Interactive effect of vaccination and vitamin D status on antibody production

| Vaccination status | First SARS-CoV-2 IgM titers | | *P* value |
| --- | --- | --- | --- |
|  | Control | Vitamin D deficiency |  |
| Unvaccinated  Routinely vaccinated  Booster vaccinated | 0.3 (0.16-0.69) n=50  0.21 (0.12-0.44) n=35  0.24 (0.15-0.61) n=39 | 0.26 (0.14-0.95) n=23  0.19 (0.12-0.41) n=31  0.26 (0.13-0.74) n=26 | 0.94  0.92  0.73 |
| *P* value | 0.37 | 0.29 |  |

| Vaccination status | Highest SARS-CoV-2 IgM titers | | *P* value |
| --- | --- | --- | --- |
|  | Control | Vitamin D deficiency |  |
| Unvaccinated  Routinely vaccinated  Booster vaccinated | 0.52 (0.27-1.49) n=50  1.1 (0.3-1.45) n=35  0.47 (0.31-1.08) n=39 | 0.47 (0.25-1.37) n=23  0.59 (0.22-0.94) n=31  0.59 (0.3-1.82) n=26 | 0.43  0.17  0.56 |
| *P* value | 0.73 | 0.44 |  |

| Vaccination status | First SARS-CoV-2 IgG titers | | *P* value |
| --- | --- | --- | --- |
|  | Control | Vitamin D deficiency |  |
| Unvaccinated  Routinely vaccinated  Booster vaccinated | 42.2 (4.1-146) n=50  9.2 (2.3-21.5) n=35  129.3 (47.4-237.9) n=39 | 71.3 (0.4-140.6) n=23  4.7 (1-19) n=31  61.7 (30.5-171.7) n=26 | 0.96  0.45  0.02 |
| *P* value | 0.03*; 0.001**; <0.001*** | 0.08*; 0.59**; <0.001*** |  |

| Vaccination status | Highest SARS-CoV-2 IgG titers | | *P* value |
| --- | --- | --- | --- |
|  | Control | Vitamin D deficiency |  |
| Unvaccinated  Routinely vaccinated  Booster vaccinated | 315 (84.3-404.8) n=50  262.9 (83.5-389.4) n=35  383.8 (339-425.9) n=39 | 263.3 (20.6-388.7) n=23  343.2 (33.6-377.3) n=31  354.9 (186.7-419.1) n=26 | 0.21  0.77  0.29 |
| *P* value | 1.00*; 0.005**; 0.008*** | 0.052 |  |

Antibody titers are presented as median (IQR). IQR, interquartile range. *P* values were adjusted with Bonferroni correction for multiple tests. SARS-CoV-2, severe acute respiratory syndrome coronavirus 2. *Unvaccinated vs. Routinely vaccinated; **Unvaccinated vs. Booster vaccinated; ***Routinely vaccinated vs. Booster vaccinated.
